# An assessment of the mathematical model for estimating of entropy optimized viscous fluid flow towards a rotating cone surface

**DOI:** 10.1038/s41598-021-89739-7

**Published:** 2021-05-13

**Authors:** Yong-Min Li, M. Ijaz Khan, Sohail A. Khan, Sami Ullah Khan, Zahir Shah, Poom Kumam

**Affiliations:** 1 Department of Mathematics, Huzhou University, Huzhou, 313000 People’s Republic of China; 2 Department of Mathematics and Statistics, Riphah International University I-14, Islamabad, 44000 Pakistan; 3 Department of Mathematics, Quaid-I-Azam University 45320, Islamabad, 44000 Pakistan; 4 Department of Mathematics, COMSATS University Islamabad, Sahiwal, 57000 Pakistan; 5 Department of Mathematical Sciences, University of Lakki Marwat, Lakki Marwat, 28420 Khyber Pakhtunkhwa Pakistan; 6 Center of Excellence in Theoretical and Computational Science (TaCS-CoE), Faculty of Science, King Mongkut’s University of Technology Thonburi (KMUTT), 126 Pracha Uthit Rd., Bang Mod, Thung Khru, Bangkok, 10140 Thailand; 7 Fixed Point Research Laboratory, Fixed Point Theory and Applications Research Group, Center of Excellence in Theoretical and Computational Science (TaCS-CoE), Faculty of Science, King Mongkut’s University of Technology Thonburi (KMUTT), 126 Pracha Uthit Rd., Bang Mod, Thung Khru, Bangkok, 10140 Thailand; 8 Department of Medical Research, China Medical University Hospital, China Medical University, Taichung, 40402 Taiwan

**Keywords:** Engineering, Mathematics and computing

## Abstract

Entropy optimization in convective viscous fluids flow due to a rotating cone is explored. Heat expression with heat source/sink and dissipation is considered. Irreversibility with binary chemical reaction is also deliberated. Nonlinear system is reduced to ODEs by suitable variables. Newton built in shooting procedure is adopted for numerical solution. Salient features velocity filed, Bejan number, entropy rate, concentration and temperature are deliberated. Numerical outcomes for velocity gradient and mass and heat transfer rates are displayed through tables. Assessments between the current and previous published outcomes are in an excellent agreement. It is noted that velocity and temperature show contrasting behavior for larger variable viscosity parameter. Entropy rate and Bejan number have reverse effect against viscosity variable. For rising values of thermal conductivity variable both Bejan number and entropy optimization have similar effect.

## Introduction

Influence of variable viscosity (temperature dependent viscosity) for flow of fluids is more realistic. Augmentation in temperature leads to decay of viscosity of liquids while gases viscosity enhances. In oiling liquids the enhancement in heat creates inner resistance which distresses the fluid viscosity, and therefore viscosity of liquid does not remain constant. Thus it is described to scrutinize the impact of different temperature variable viscosity. Mukhopadhyay and Layek^1^ studied the radiative convective flow by a porous stretchable surface with temperature dependant viscosity. Impact of variable viscosity in an unsteady magnetohydrodynamic convection flow is investigated by Seddeek^2^. Salient features of variable properties for thin film flow is explored by Khan et al.^3^. Hayat et al.^4^ studied unsteady convective viscous liquids flow. Effect of heat flux on unsteady magnetohydrodynamic viscous liquids flow over a rotating disk is discoursed by Turkyilmazoglu^5^. Hayat et al.^6^ scrutinized the behavior of chemical reaction in Jeffrey liquid flow with variable thermal conductivity. Some relevant attempts about variable properties made in Refs.^7–10^.

The ability of noteworthy improvement apparatus such as spinning cone columns, centrifugal disc atomizers, fluid degausser, rotating packed-bed reactors and centrifugal film evaporators etc. depends upon the nature of motion of liquid and pressure distributions. Rotating cone has utilizations in engineering field, advanced nanotechnology and industrial sites including nuclear reactor, liquid film evaporators and cooling system etc. Shevchuk^11^ successfully presented the novel numerical and analytical simulations for the various rotating flows like system rotation, swirl flows associated with the swirl generators and surface curvature in bends as well as turns. The impact of centrifugal and Coriolis forces on the distinct flow pattern due to rotating flows was also successfully presented in this scientific continuation. The work of Shevchuk^12^ visualized the impact of wall temperature in order to inspect the heat transfer characteristics in the laminar flow confined by rotating disk. The analytical solutions for the formulated rotating disk problems were also successfully addressed. In interesting another continuation, Shevchuk^13^ modeled the turbulent flow problem in presence of heat transfer phenomenon due to rotating disk. The applications of heat and mass transfer pattern in rotating flow of cone and plate devices has been pointed out by Shevchuk^14^. Turkyilmazoglu^15^ presented the analytical solutions for a rotating cone problem for viscous fluid. In another continuation, Turkyilmazoglu^16^ inspected the heat transfer pattern in viscous fluid confined by a rotating cone. Behaviors of variable properties on mixed convection viscous liquid flow with dissipation over a rotating cone are deliberated by Malik et al.^17^. Turkyilmazoglu^18^ analyzed the fluctuation in heat transfer mechanism for viscous fluid flow configured by rotating disk in with porous space. Impact of variable viscosity in magnetohydrodynamic flow of Carreau nanofluid by a rotating cone is illustrated by Ghadikolaei et al.^19^. Sulochana et al.^20^ studied radiative magnetohydrodynamic flow of laminar liquid with Soret effect over a rotating cone. Salient behaviors of thermal flux in unsteady MHD convective flow due to a rotating cone are presented by Osalusi et al.^21^. Turkyilmazoglu^22^ addressed the radially impacted flow of viscous fluid accounted by rotating disk. Asghar et al.^23^ used Lie group approach to simulate the solution for a rotating flow problem in presence of heat transfer. Turkyilmazoglu^24^ visualized the flow pattern of triggered fluid due to rotating stretchable disk. The fluid flow due to stationary and moving rotating cone subject to the magnetic force impact has been depicted by Turkyilmazoglu^25^.

With excellent thermal effectiveness and multidisciplinary applications, the study of nanoparticles becomes the dynamic objective of scientists. The valuable importance of nano-materials in distinct processes includes solar systems, technological processes, engineering devices, nuclear reactors, cooling phenomenon etc. With less than 100 nm size and structure, the nanoparticles are famous due to extra-ordinary thermal performances in contrast to base liquids. In modern medical sciences, the nanoparticles are used to demolish the precarious cancerous tissues. Choi^26^ presents the novel investigation on nanofluids and examined the extra-ordinary thermal activities of such materials. Later on, many investigations are claimed in the literature to analyze the thermal assessment of nano-materials. For example, Chu^27^ explained the thermal aspects of third grade nanofluid with significances of activation energy and microorganisms. Majeed et al.^28^ inspected the improvement in thermal properties of conventional base fluids with interaction of magnetic nano-fluid subject to the dipole effects. Hassan et al.^29^ visualized the shape factor in ferrofluid with dynamic of oscillating magnetic force. The thermal inspection in Maxwell nanofluid with external impact of heat generation was directed numerically by Majeed et al.^30^. Khan^31^ discussed the entropy optimized flow of hybrid nanofluid over a stretched surface of rotating disk. The enhanced features of metallic nanoparticles subject to the magnetic dipole phenomenon were addressed by Majeed et al.^32^.

In microscopic level the entropy rate is caused due to heat transfer, molecular vibration, dissipation, spin movement, molecular friction, kinetic energy Joule heating etc. and heat loss occurs. For improvement the productivity of numerous thermal schemes, it is necessary to optimize the irreversibility. Thermodynamic second law redirects more significant behaviors in comparison to thermodynamic first law. Thermodynamics second law gives the entropy optimization and scientific tools for decrease of confrontation. It helps us to develop the ability of various engineering improvements. These processes encompass heat conduction and furthermore to calculate the entropy generation rate. Primary attention of entropy generation problems is done by Bejan^33^. Zhou et al.^34^ discussed irreversibility analysis about convective flow of nanoliquids in a cavity. Salient characteristics of thermophoretic and Brownian diffusion in flow of Prandtl-Eyring liquids with entropy optimization are exemplified by Khan et al.^35^. Irreversibility analysis in magnetohydrodynamic flow of Carreau nanofluids through Buongiorno nanofluid model is validated by Khan et al.^36^. Jiang and Zhou^37^ studied viscous nanoliquid flow with irreversibility. Some advancement about irreversibility analysis is given in Refs.^38–45^.

The above presented research work, it is observed that no determination has been completed to investigate the irreversibility consideration for convective viscous fluid flow over a rotating cone. Therefore intension in this paper is to scrutinize the irreversibility for mixed convection reactive flow of viscous fluid by a rotating cone. Heat transfer is demonstrated with heat generation/absorption and dissipation. Furthermore a physical characteristic of entropy is considered. Nonlinear governing system is altered to ODEs. The given system is than tackled through NDSolve procedure. Prominent characteristics of different engineering variable on velocity field, entropy rate, Bejan number, concentration and temperature are realistically examined. The computational outcomes of surface drag force, heat transfer rate and gradient of concentration are scrutinized via different remarkable parameters.

## Formulation

**Figure 1:**
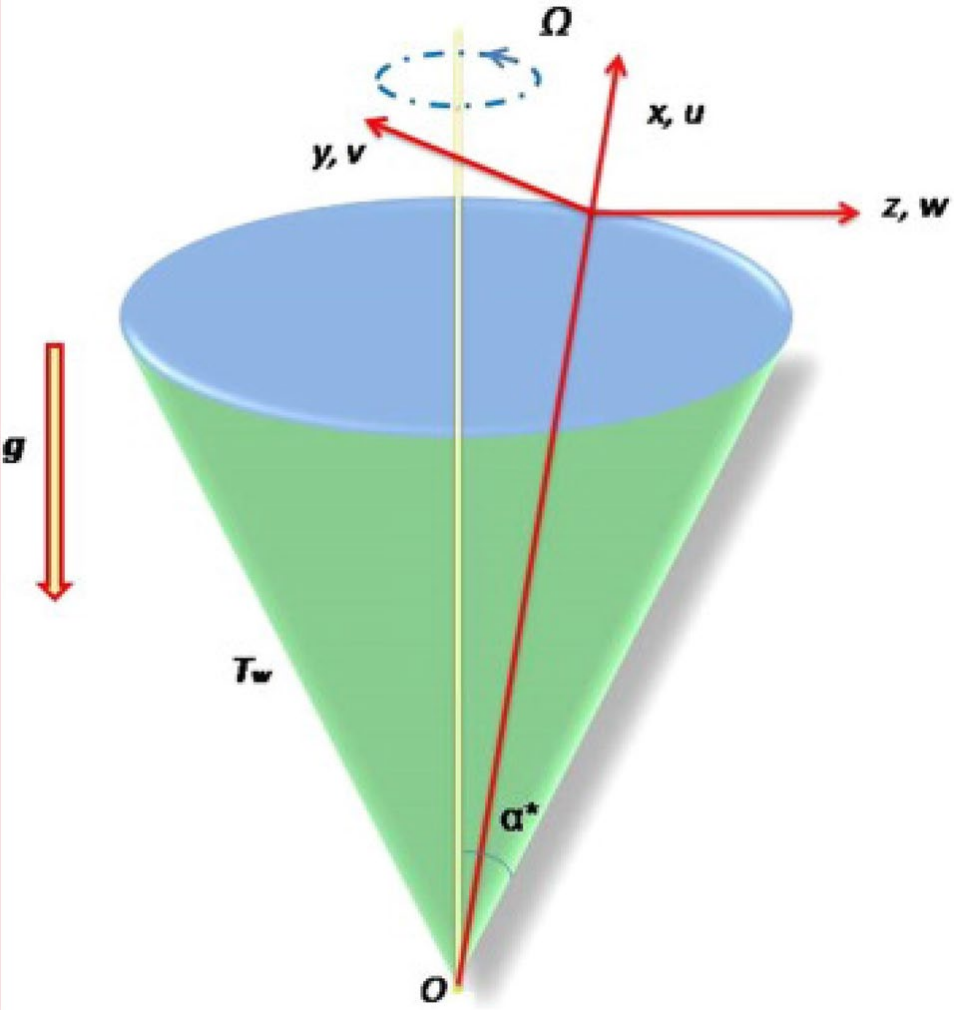
Sketch of problem^9,10^.

We examine mixed convective flow of incompressible laminar fluid over a rotating cone. Angular velocity is denoted by $\left( \Omega \right)$. Energy expression with heat source/sink and dissipation is considered. Innovative behaviors regarding entropy optimization is accounted. First order chemical reaction is deliberated. The resistive force arises owing to variation in concentration and temperature in the liquid and flow is axi-symmetric. The acceleration associated with gravitational force are assumed along the downward direction. Figure 1 describes the physical model^9,10^.

The related expressions are^15,16^:

1 $$ \frac{{\partial \left( {xu} \right)}}{\partial x} + \frac{{\partial \left( {xw} \right)}}{\partial z} = 0,\, $$

2 $$ u\frac{\partial u}{{\partial x}} - \frac{{v^{2} }}{x} + w\frac{\partial u}{{\partial z}} = \frac{1}{\rho }\frac{\partial }{\partial z}\left( {\mu \left( T \right)\,\frac{\partial u}{{\partial z}}} \right) + g\beta_{t} \cos \alpha^{ * } \left( {T - T_{\infty } } \right) + g\beta_{c} \cos \alpha^{ * } \left( {C - C_{\infty } } \right),\, $$

3 $$ u\frac{\partial v}{{\partial x}} + \frac{uv}{x} + w\frac{\partial v}{{\partial z}} = \frac{1}{\rho }\frac{\partial }{\partial z}\left( {\mu \left( T \right)\,\frac{\partial v}{{\partial z}}} \right),\, $$

4 $$ u\frac{\partial T}{{\partial x}} + w\frac{\partial T}{{\partial z}} = \frac{1}{{\rho c_{p} }}\frac{\partial }{\partial z}\left( {k\left( T \right)\,\frac{\partial T}{{\partial z}}} \right) + \frac{\mu \left( T \right)}{{\rho c_{p} }}\left[ {\left( {\frac{\partial u}{{\partial z}}} \right)^{2} + \left( {\frac{\partial v}{{\partial z}}} \right)^{2} } \right] + \frac{{Q_{0} }}{{\rho c_{p} }}\left( {T - T_{\infty } } \right),\, $$

5 $$ u\frac{\partial C}{{\partial x}} + w\frac{\partial C}{{\partial z}} = D_{B} \frac{{\partial^{2} C}}{{\partial z^{2} }} - k_{r} \left( {C - C_{\infty } } \right), $$

with

6 $$ \left. \begin{gathered} u = 0, \, v = \Omega x\sin \alpha^{ * } {, }w = 0{, }T = T_{w} {, }C = C_{w} \, at \, z = 0 \hfill \\ u = 0{, }v = 0{, }T = T_{\infty } {, }C = C_{\infty } \, as \, z \to \infty \hfill \\ \end{gathered} \right\} $$

here viscosity and conductivity are employed in the forms^44^

7 $$ \mu = \mu_{0} e^{{ - \zeta \left( {T - T_{\infty } } \right)}} ,\, $$

8 $$ \mu = \mu_{0} \left( {1 - A\theta } \right)\,{\text{, where }}A = \zeta \left( {T_{w} - T_{\infty } } \right). $$

9 $$ k = k_{0} e^{{ - c\left( {T - T_{\infty } } \right)}} ,\, $$

10 $$ k = k_{0} \left( {1 + \delta \theta } \right){\text{, where }}\delta = - c\left( {T_{w} - T_{\infty } } \right) $$

here $\rho$ denotes the density, $\mu_{0}$ the constant viscosity, $u,v$ and $w$ the velocity components, $\alpha^{ * }$ the semi-vertical angle, $\beta_{c}$ the coefficient of solutal expansion, $A$ the variable viscosity parameter, $\beta_{t}$ the thermal coefficient expansion, $T$, the temperature, $k_{0}$ the constant thermal conductivity, $c_{p}$ the specific heat, $T_{w}$ the wall temperature, $\delta$ the variable thermal conductivity parameter, $T_{\infty }$ the ambient temperature, $Q_{0}$ the heat generation/absorption coefficient, $C$ the concentration, $\Omega$ the dimensionless angular velocity, $C_{\infty }$ the ambient concentration $D_{B}$ the mass diffusivity, $C_{w}$ the wall concentration and $k_{r}$ the chemical reaction rate.

Letting

11 $$ \left. {\begin{array}{*{20}c} {u = - \tfrac{1}{2}\Omega x\sin \alpha^{ * } f^{\prime}\left( \eta \right), \, v = \Omega x\sin \alpha^{ * } g\left( \eta \right), \, w = \left( {\nu_{0} \Omega \sin \alpha^{ * } } \right)^{1/2} f\left( \eta \right),} \\ {\theta \left( \eta \right) = \tfrac{{(T - T_{\infty } )}}{{(T_{w} - T_{\infty } )}}, \, (T_{w} - T_{\infty } ) = (T_{0} - T_{\infty } )\left( {\tfrac{x}{L}} \right), \, \phi \left( \eta \right) = \tfrac{{(C - C_{\infty } )}}{{(C_{w} - C_{\infty } )}},} \\ {(C_{w} - C_{\infty } ) = (C_{0} - C_{\infty } )\left( {\tfrac{x}{L}} \right), \, \eta = \left( {\tfrac{{\Omega \sin \alpha^{ * } }}{{\nu_{0} }}} \right)^{1/2} z} \\ \end{array} } \right\} $$

one has

12 $$ \left( {1 - A\theta } \right)f^{\prime\prime\prime} - A\theta^{\prime}f^{\prime\prime} + \frac{1}{2}f^{{\prime}{2}} - 2g^{2} - ff^{\prime\prime} - 2\lambda \left( {\theta + N\phi } \right) = 0, $$

13 $$ \left( {1 - A\theta } \right)\,g^{\prime\prime} - A\theta^{\prime}g^{\prime} + f^{\prime}g - fg^{\prime} = 0,\, $$

14 $$ \left( {1 + \varepsilon \theta } \right)\,\theta^{\prime\prime} - \mathop {\Pr }\limits f\theta^{\prime} + \varepsilon \theta^{{\prime}{2}} \frac{1}{2}\mathop {\Pr }\limits f^{\prime}\theta + \mathop {\Pr }\limits Ec\left( {1 - A\theta } \right)\,\left( {\frac{1}{4}f^{{\prime\prime}{2}} + g^{{\prime}{2}} } \right) + \mathop {\Pr }\limits \beta \theta = 0,\, $$

15 $$ \phi^{\prime\prime}\frac{1}{2}Scf^{\prime}\phi - Scf\phi^{\prime} - \gamma Sc\phi = 0,\, $$

16 $$ \left. {\begin{array}{*{20}c} {f\left( 0 \right) = 0,\, \, f^{\prime}\left( 0 \right) = 0,\, \, g\left( 0 \right) = 1,\, \, \theta \left( 0 \right) = 1,\, \, \phi \left( 0 \right) = 1} \\ {f^{\prime}\left( \infty \right) = 0,\, \, g\left( \infty \right) = 0,\, \, \theta \left( \infty \right) = 0,\, \, \phi \left( \infty \right) = 0} \\ \end{array} } \right\}\, $$

where $\lambda \left( { = \tfrac{Gr}{{Re^{2} }}} \right)$ shows the mixed convection parameter, $Re\left( { = \tfrac{{L^{2} \Omega \sin \alpha^{ * } }}{\nu }} \right)$ the Reynold number, $Gr\left( { = \tfrac{{g\beta_{t} \cos \alpha^{ * } \left( {T_{0} - T_{\infty } } \right)\,L^{3} }}{{\nu^{2} }}} \right)$ the Grashoff number, $N\left( { = \tfrac{{\beta_{c} \left( {C_{0} - C_{\infty } } \right)}}{{\beta_{t} \left( {T_{0} - T_{\infty } } \right)}}} \right)$ the buoyancy ratio variable, $Ec\left( { = \tfrac{{\Omega^{2} Lx\sin^{2} \alpha^{ * } }}{{c_{p} \left( {T_{0} - T_{\infty } } \right)}}} \right)$ the Eckert number, $\Pr \left( { = \tfrac{{\nu_{0} }}{\alpha }} \right)$ the Prandtl number, $\beta \left( { = \tfrac{{Q_{0} }}{{\left( {\rho c_{p} } \right)\,\Omega \sin \alpha^{ * } }}} \right)$ the heat generation variable, $\gamma \left( { = \tfrac{{k_{r} }}{{\Omega \sin \alpha^{ * } }}} \right)$ the chemical reaction variable and $Sc\left( { = \tfrac{{\nu_{0} }}{D}} \right)$ the Schmidt number.

## Entropy modeling

Mathematically entropy optimization is given by^41–43^:

17 $$ S_{G} = \frac{k\left( T \right)}{{T_{\infty }^{2} }}\left( {\frac{\partial T}{{\partial z}}} \right)^{2} + \frac{\mu \left( T \right)}{{T_{\infty } }}\left[ {\left( {\frac{\partial u}{{\partial z}}} \right)^{2} + \left( {\frac{\partial v}{{\partial z}}} \right)^{2} } \right] + \frac{{R_{D} }}{{T_{\infty } }}\left( {\frac{\partial T}{{\partial z}}\frac{\partial C}{{\partial z}}} \right) + \frac{{R_{D} }}{{C_{\infty } }}\left( {\frac{\partial C}{{\partial z}}} \right)^{2} $$

while after utilization of Eq. (11) yields^41–43^:

18 $$ N_{G} = \alpha_{1} \left( {1 + \varepsilon \theta } \right)\,\theta^{{\prime}{2}} + \frac{Br}{{A_{1} }}\left( {1 - A\theta } \right)\,\left( {\frac{1}{4}f^{{\prime\prime}{2}} + g^{{\prime}{2}} } \right) + L\theta^{\prime}\phi^{\prime} + L\frac{{\alpha_{2} }}{{\alpha_{1} }}\phi^{{\prime}{2}} $$

Bejan number is given as^41–43^:

19 $$ Be = \frac{{\text{Thermal and solutal transfer irreversibility}}}{{\text{Total irreversibility}}},\, $$

or

20 $$ Be = \frac{{\alpha_{1} \left( {1 + \varepsilon \theta } \right)\,\theta^{{\prime}{2}} + L\theta^{\prime}\phi^{\prime} + L\tfrac{{\alpha_{2} }}{{\alpha_{1} }}\phi^{{\prime}{2}} }}{{\alpha_{1} \left( {1 + \varepsilon \theta } \right)\,\theta^{{\prime}{2}} + \tfrac{Br}{{A_{1} }}\left( {1 - A\theta } \right)\,\left( {\tfrac{1}{4}f^{{\prime\prime}{2}} + g^{{\prime}{2}} } \right) + L\theta^{\prime}\phi^{\prime} + L\tfrac{{\alpha_{2} }}{{\alpha_{1} }}\phi^{{\prime}{2}} }} $$

in which $N_{G} \left( { = \tfrac{{\nu_{0} S_{G} T_{\infty } L^{2} }}{{k_{0} \Omega x^{2} \sin \alpha^{ * } \left( {T_{0} - T_{\infty } } \right)}}} \right)$ signifies the entropy rate, $Br\left( { = \tfrac{{\mu_{0} \Omega^{2} xL\sin \alpha^{ * } }}{{k_{0} \left( {T_{0} - T_{\infty } } \right)}}} \right)$ the Brinkman number, $\alpha_{2} \left( { = \tfrac{{C_{0} - C_{\infty } }}{{C_{\infty } }}} \right)$ the concentration ratio parameter, $\alpha_{1} \left( { = \tfrac{{\left( {T_{0} - T_{\infty } } \right)}}{{T_{\infty } }}} \right)$ the temperature difference variable, $A\left( { = \tfrac{x}{L}} \right)$ dimensionless parameter and $L\left( { = \tfrac{{R_{D} \left( {C_{0} - C_{\infty } } \right)}}{k}} \right)$ the diffusion variable.

## Physical quantities

### Velocity gradient

Surface drag forces $\left( {C_{fx} {\text{ and }}C_{fy} } \right)$ are given as

21 $$ C_{fx} = \frac{{\left. {2\tau_{xz} } \right|_{z = 0} }}{{\rho \left( {\Omega x\sin \alpha^{ * } } \right)^{2} }}{, }C_{fy} = \frac{{\left. {2\tau_{yz} } \right|_{z = 0} }}{{\rho \left( {\Omega x\sin \alpha^{ * } } \right)^{2} }},\, $$

with $\tau_{xz}$ and $\tau_{yz}$ as shear stresses are given by

22 $$ \tau_{xz} = \mu \left( T \right)\,\left( {\frac{\partial u}{{\partial z}}} \right)\,{, }\tau_{yz} = \mu \left( T \right)\,\left( {\frac{\partial v}{{\partial z}}} \right),\, $$

Finally we can write

23 $$ C_{fx} Re_{x}^{1/2} = - \left( {1 - A\theta } \right)\,f^{\prime\prime}\left( 0 \right)^{2} {, }\frac{1}{2}C_{fy} Re_{x}^{1/2} = - \left( {1 - A\theta } \right)\,g^{\prime}\left( 0 \right)^{2} . $$

### Nusselt number

It is expressed as

24 $$ Nu_{x} = \frac{{\left. {xq_{w} } \right|_{z = 0} }}{{\left( {T_{w} - T_{\infty } } \right)}},\, $$

with heat flux $q_{w}$ represented by

25 $$ q_{w} = - \left( {\frac{\partial T}{{\partial z}}} \right),\, $$

now

26 $$ Nu_{x} Re_{x}^{ - 1/2} = - \theta^{\prime}\left( 0 \right). $$

### Mass transfer rate

Sherwood number $\left( {Sh_{x} } \right)$ is

27 $$ Sh_{x} = \frac{{\left. {xh_{w} } \right|_{z = 0} }}{{\left( {C_{w} - C_{\infty } } \right)}},\, $$

with $h_{w}$ as mass flux through following expression

28 $$ h_{w} = - \left( {\frac{\partial C}{{\partial z}}} \right),\, $$

Finally we have

29 $$ Sh_{x} Re_{x}^{ - 1/2} = - \phi^{\prime}\left( 0 \right). $$

## Validation of results

Tables 1 and 2 are provided to authenticate the precision of current outcome with aforementioned published outcomes in literature. These tables deliberated the evaluation of velocity gradient and Nusselt number versus increasing values of $\left( \lambda \right)$ with those of Saleem and Nadeem^34^ and Chamka et al.^35^. These outcomes are established in good agreement.

**Table 1:** Comparison of surface drag force with Saleem and Nadeem^44^ and Chamka et al.^45^.

$\Pr$	$\lambda$	Saleem and Nadeem^44^		Chamka et al.^45^		Recent results	
		$C_{fx} Re_{x}^{1/2}$	$\tfrac{1}{2}C_{fy} Re_{x}^{1/2}$	$C_{fx} Re_{x}^{1/2}$	$\tfrac{1}{2}C_{fy} Re_{x}^{1/2}$	$C_{fx} Re_{x}^{1/2}$	$\tfrac{1}{2}C_{fy} Re_{x}^{1/2}$
0.7	0.0	1.0255	0.6154	1.0255	0.6158	1.0255	0.6156
	1.0	2.2010	0.8493	2.2012	0.8496	2.2010	0.8494
	10.0	8.5042	1.3992	8.5041	1.3995	8.5043	1.3992
10.0	0.0	1.0255	0.6158	1.0256	0.6158	1.0256	0.6158
	1.0	1.5630	0.6835	1.5636	0.6837	1.5631	0.6835
	10.0	5.0820	0.9845	5.0821	0.9840	5.0822	0.9842

**Table 2:** Comparison of Nusselt number with Saleem and Nadeem^44^ and Chamka et al.^45^.

$\Pr$	$\lambda$	Saleem and Nadeem^44^	Chamka et al.^45^	Recent results
0.7	0.0	0.4299	0.4299	0.4298
	1.0	0.6121	0.6120	0.6122
	10.0	1.3992	1.0097	1.3993
10.0	0.0	1.4111	1.4110	1.4119
	1.0	1.5661	1.5662	1.5664
	10.0	2.3581	2.3580	2.3583

## Physical description

Noticeable performances of various sundry variables about entropy rate, temperature, velocity field, Bejan number and concentration and are deliberated through graphs. Velocity gradient and Nusselt and Sherwood numbers are numerically computed against various parameters. The analysis is performed for flow parameters with specified numerical values range like $0.1 \le A \le 1.5,$ $0.2 \le N \le 1.5,$ $0.1 \le \beta \le 0.9,$ $0.3 \le \lambda \le 0.9,$ $1 \le Sc \le 3,$ $0.2 \le L \le 0.8,$ $0.5 \le \Pr \le 1.5,$ $0.2 \le \gamma \le 1.6$ and $0.2 \le Br \le 1.4.$

### Velocity

**Figure 2:**
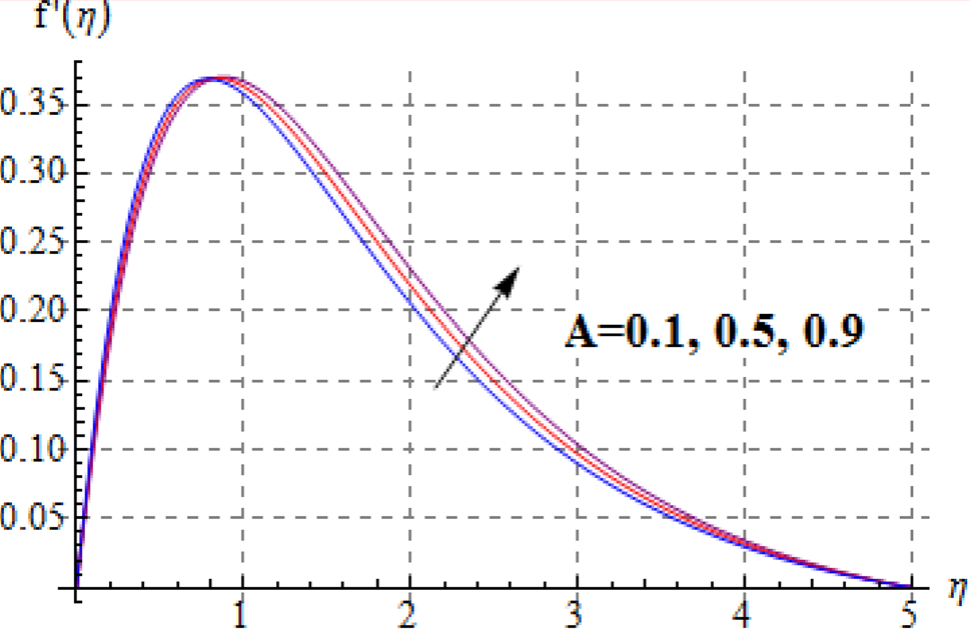
$$f^{\prime}\left( \eta \right)$$ against A.

**Figure 3:**
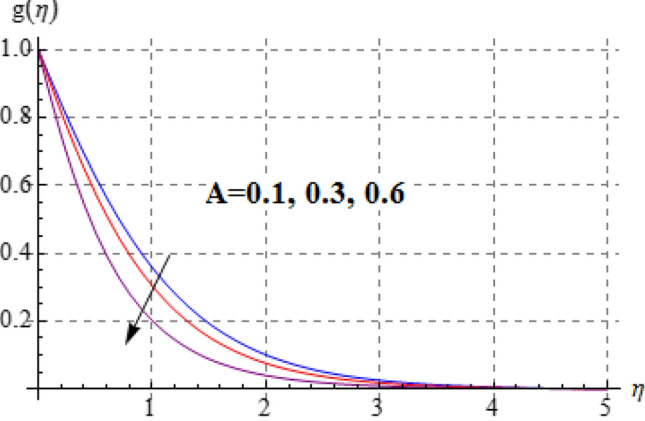
$$g\left( \eta \right)$$ against A.

**Figure 4:**
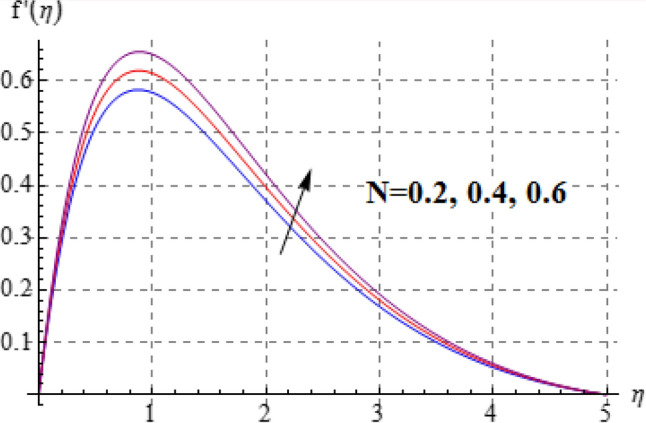
$$f^{\prime}\left( \eta \right)$$ against N.

**Figure 5:**
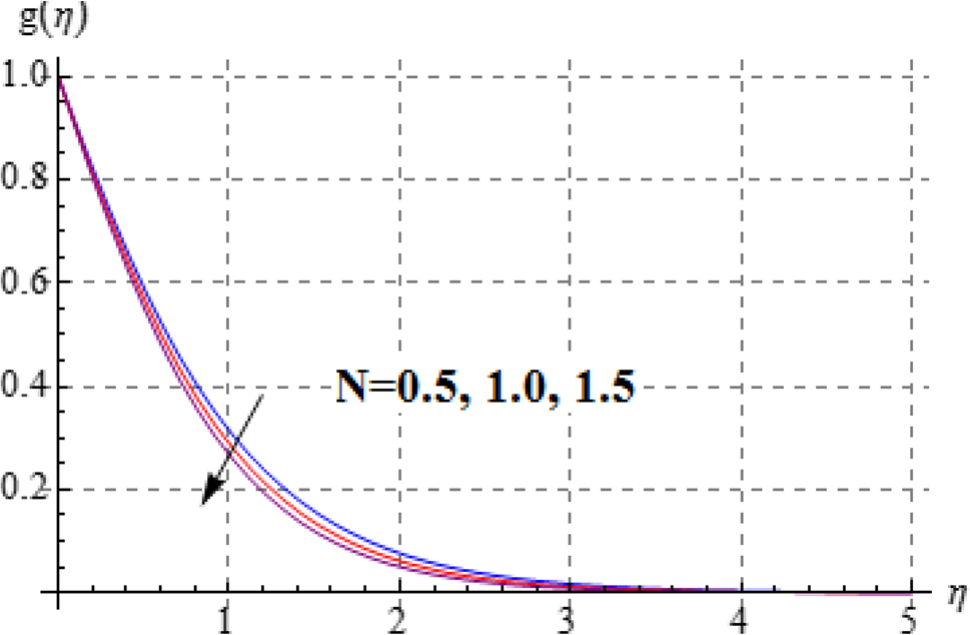
$$g\left( \eta \right)$$ against N.

**Figure 6:**
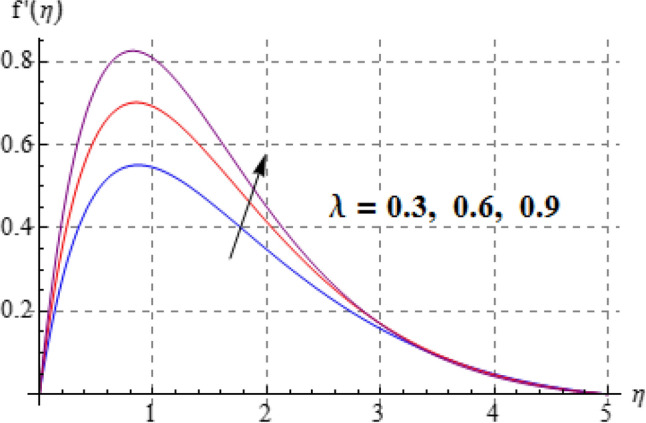
$$f^{\prime}\left( \eta \right)$$ against $$\lambda$$.

**Figure 7:**
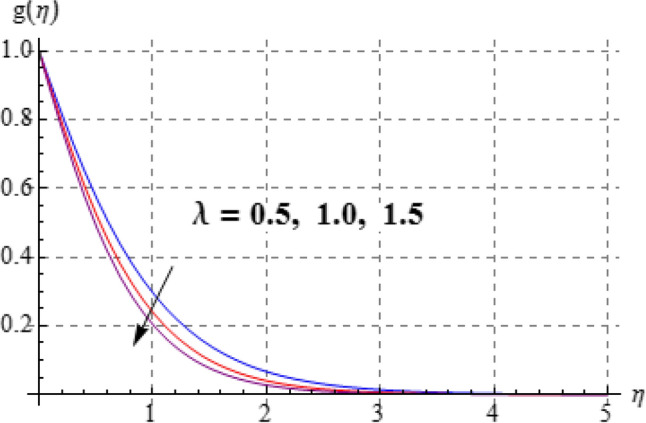
$$g\left( \eta \right)$$ against $$\lambda$$.

Salient effects of $\left( A \right)$, $\left( N \right)$ and $\left( \lambda \right)$ on $f^{\prime}\left( \eta \right)$ (tangential velocity) and $g\left( \eta \right)$ (azimuthal velocity) are examined in Figs. 2, 3, 4, 5, 6 and 7. Figure 2 depicts characteristics of tangential velocity $\left( {f^{\prime}\left( \eta \right)} \right)$ for viscosity parameter $\left( A \right)$. For increasing values of $\left( A \right)$ an enhancement occurs in $f^{\prime}\left( \eta \right)$. Characteristic of $\left( A \right)$ on $g\left( \eta \right)$ is exposed in Fig. 3. Clearly $g\left( \eta \right)$ is a decaying function of viscosity parameter $\left( A \right)$. In fact increments in $\left( A \right)$ leads to reduction in temperature difference (convective potential) between ambient fluid heated surface and as a result azimuthal velocity $\left( {g\left( \eta \right)} \right)$ decays. Figures 4 and 5 scrutinize the behaviors of $\left( N \right)$ on $f^{\prime}\left( \eta \right)$ (tangential velocity) and $g\left( \eta \right)$ (azimuthal velocity). One can find that $f^{\prime}\left( \eta \right)$ and $g\left( \eta \right)$ have reverse effects via larger $\left( N \right)$. In fact augmentation in $\left( N \right)$ makes the fluid viscous and consequently $g\left( \eta \right)$ decreases. Characteristics of $\left( \lambda \right)$ on $f^{\prime}\left( \eta \right)$ and $g\left( \eta \right)$ are demonstrated in Figs. 6 and 7. These figures demonstrates that higher estimation of $\left( \lambda \right)$ improves the tangential velocity $\left( {f^{\prime}\left( \eta \right)} \right)$, while reverse effect holds for azimuthal velocity $\left( {g\left( \eta \right)} \right)$.

### Temperature

**Figure 8:**
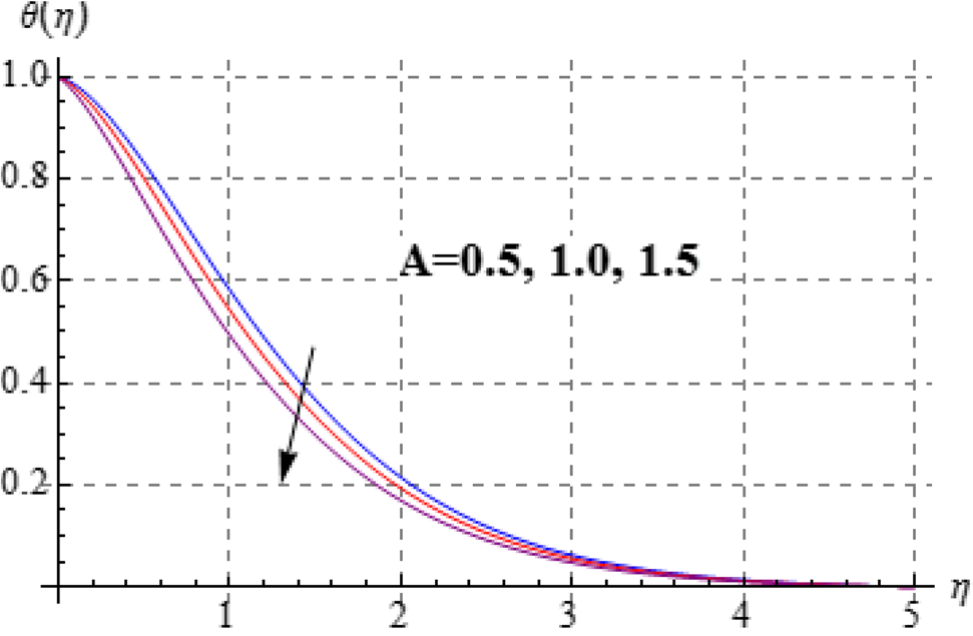
$$\theta \left( \eta \right)$$ against A.

**Figure 9:**
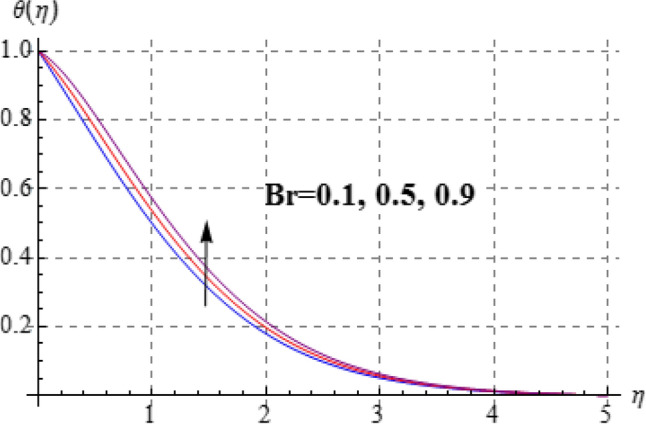
$$\theta \left( \eta \right)$$ against Br.

**Figure 10:**
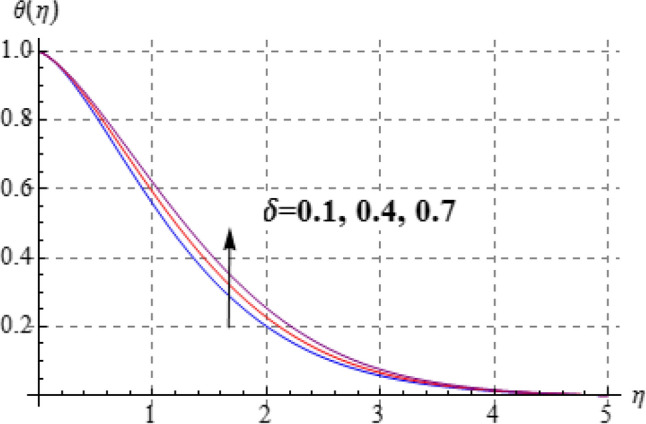
$$\theta \left( \eta \right)$$ against $$\delta$$.

**Figure 11:**
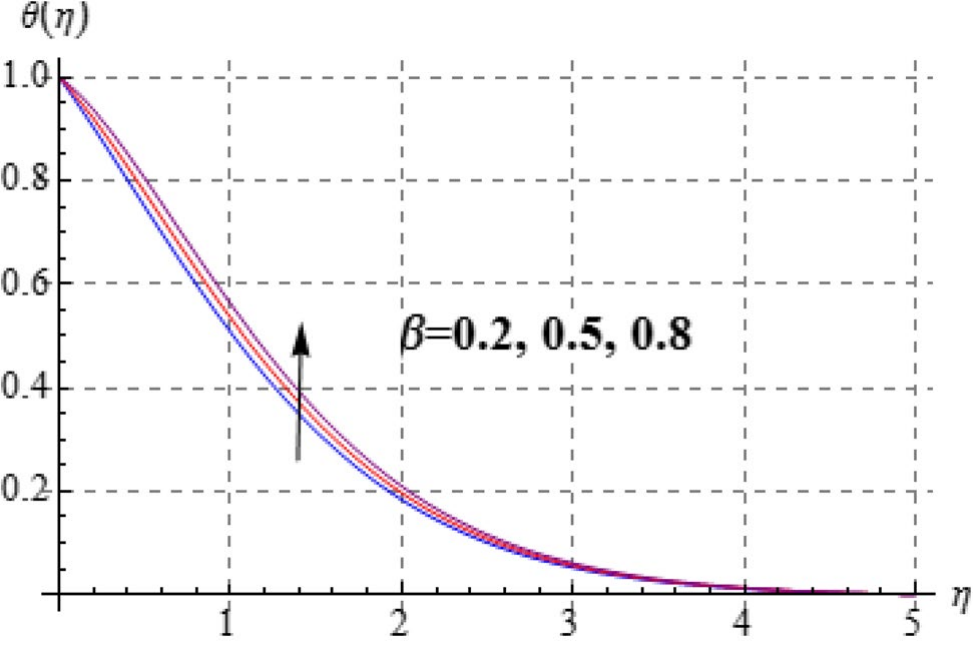
$$\theta \left( \eta \right)$$ against $$\beta$$.

**Figure 12:**
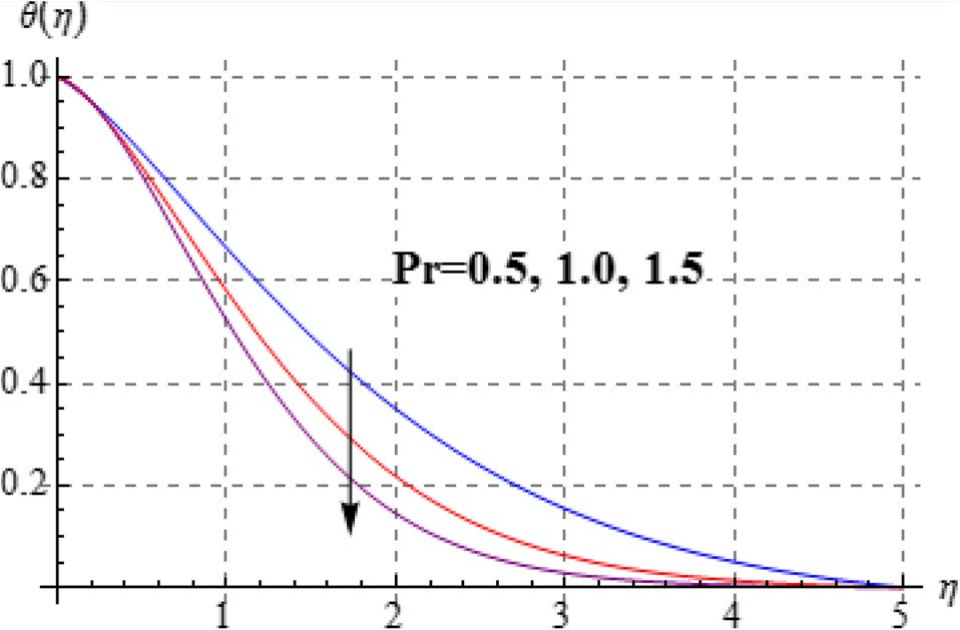
$$\theta \left( \eta \right)$$ against $$\Pr$$.

Figures 8, 9, 10, 11 and 12 have been displayed to explore behavior of pertinent variables like $\left( A \right)$, $\left( {Br} \right)$, $\left( \delta \right)$, $\left( \beta \right)$ and $\left( {\Pr } \right)$ on $\theta \left( \eta \right)$. Figure 8 studied effect of viscosity variable $\left( A \right)$ on $\left( {\theta \left( \eta \right)} \right)$. Clearly temperature is a decreasing function of $\left( A \right)$. Outcome of (Br) on temperature is sketched in Fig. 9. Here the increasing values of $\left( {Ec} \right)$ corresponds to an augmentation in $\theta \left( \eta \right)$. For larger Brinkman number the slower heat transmission is produced by viscous force and therefore $\theta \left( \eta \right)$ boosts up. Figure 10 interprets the behaviors of $\left( \delta \right)$ on temperature. We noted that temperature improves through $\left( \delta \right)$. Variation of $\left( \beta \right)$ on $\theta \left( \eta \right)$ is interpreted in Fig. 11. Temperature $\left( {\theta \left( \eta \right)} \right)$ against $\left( \beta \right)$ rises. Figure 12 is devoted to see the outcome of $\left( {\Pr } \right)$ on $\theta \left( \eta \right)$. Clearly larger $\left( {\Pr } \right)$ the thermal layer reduces which improves and heat transfer rate improves. Therefore $\theta \left( \eta \right)$ decays.

### Concentration

**Figure 13:**
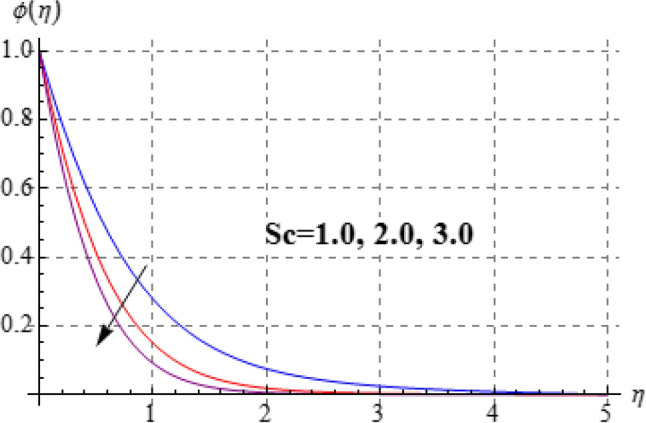
$$\theta \left( \eta \right)$$ against $$Sc$$.

**Figure 14:**
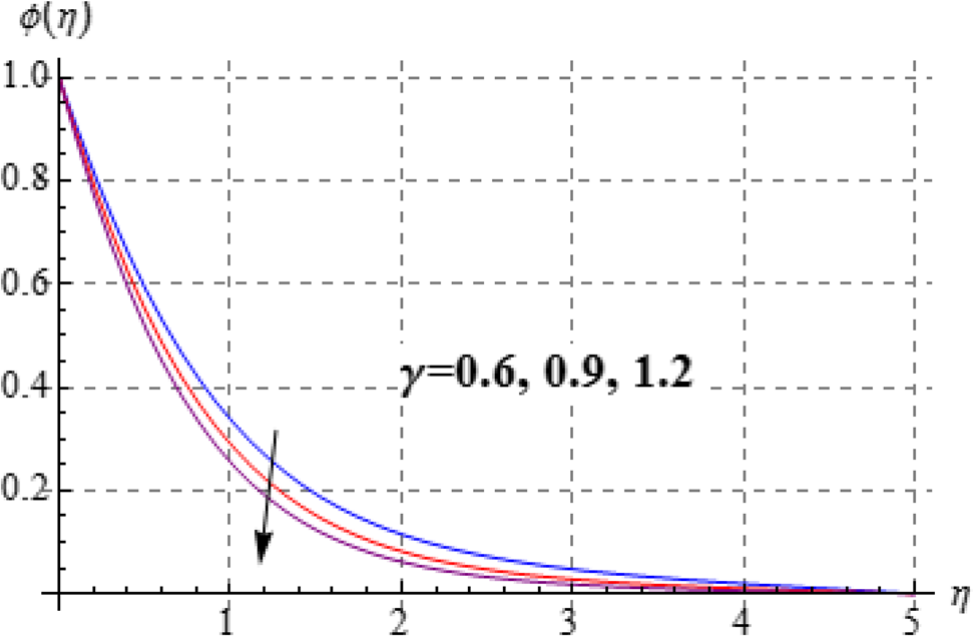
$$\theta \left( \eta \right)$$ against $$\gamma$$.

Impact of $\left( {Sc} \right)$ on $\phi \left( \eta \right)$ is plotted in Fig. 13. Through Schmidth number, the concentration decays. Figure 14 is depicts the characteristics of $\left( \gamma \right)$ on concentration $\left( {\phi \left( \eta \right)} \right)$. Clearly $\phi \left( \eta \right)$ is diminished for higher estimation of $\left( \gamma \right)$. The fluid acts thick for higher $\left( \gamma \right)$ and so reduction in $\phi \left( \eta \right)$ occurs.

### Entropy and Bejan number

**Figure 15:**
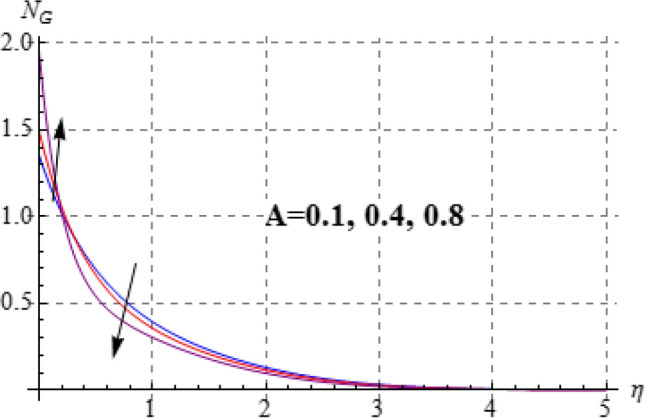
$$N_{G}$$ against A.

**Figure 16:**
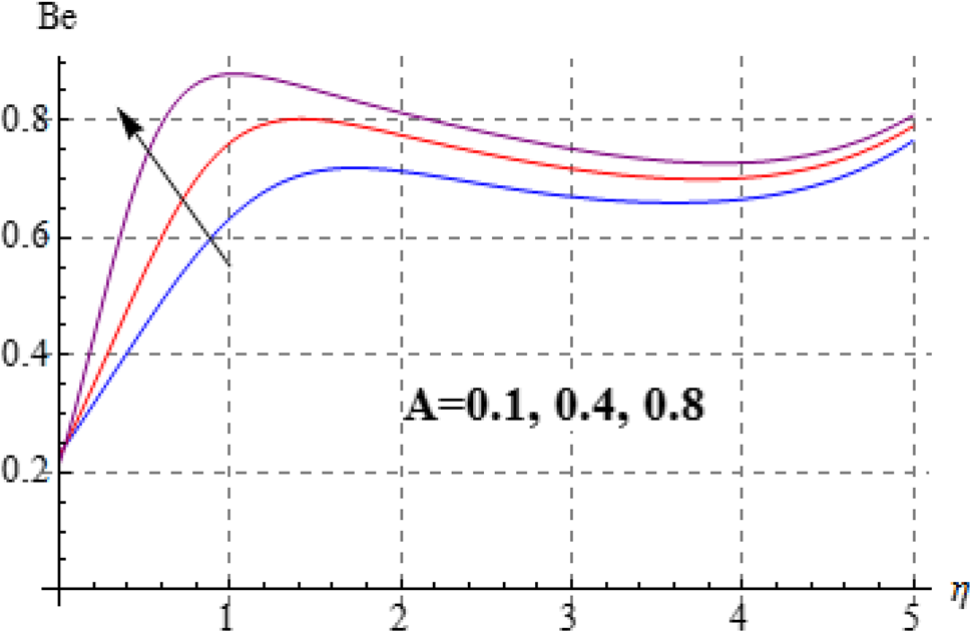
$$Be$$ against A.

**Figure 17:**
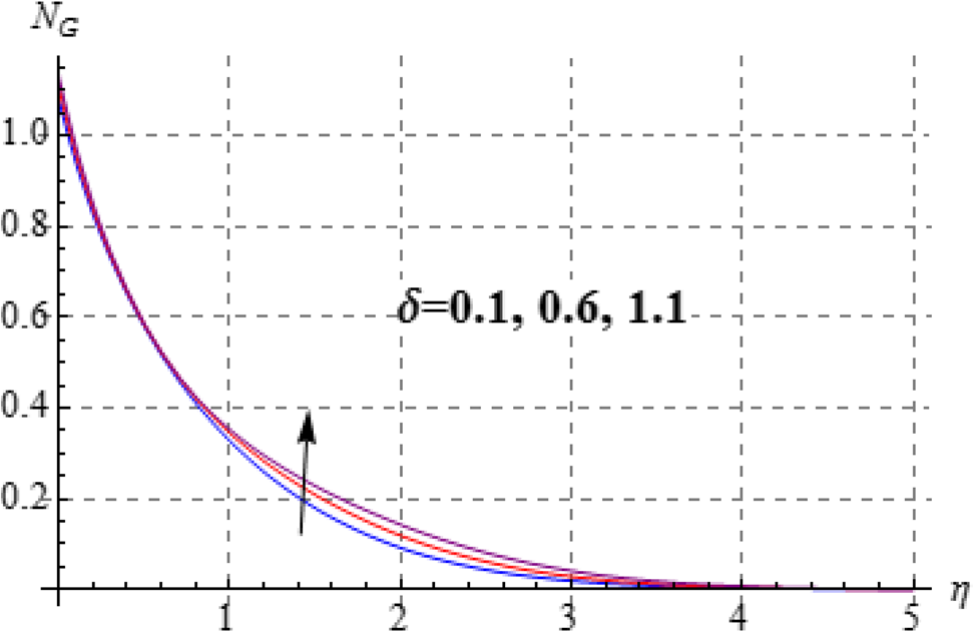
$$N_{G}$$ against $$\delta$$.

**Figure 18:**
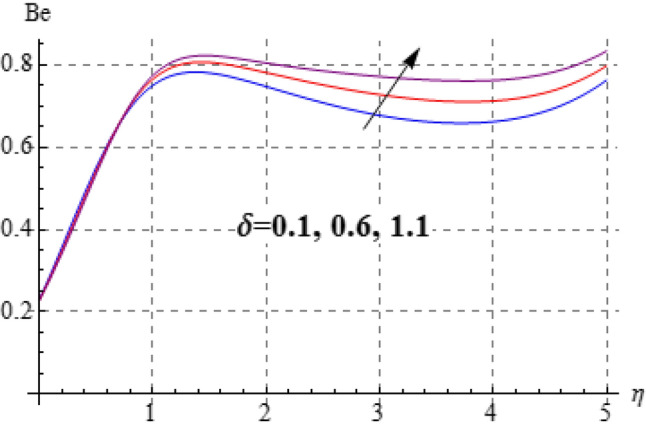
$$N_{G}$$ against $$\delta$$.

**Figure 19:**
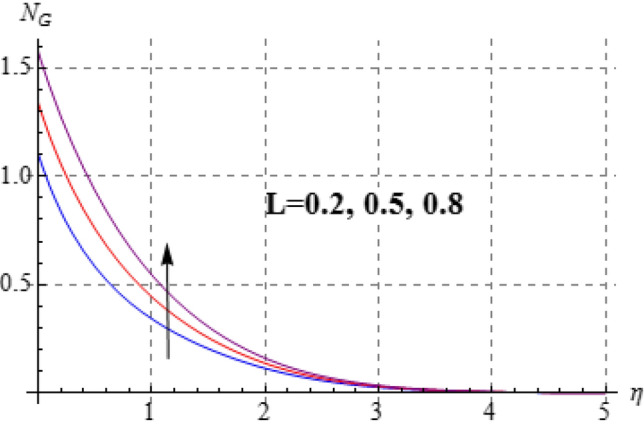
$$N_{G}$$ against L.

**Figure 20:**
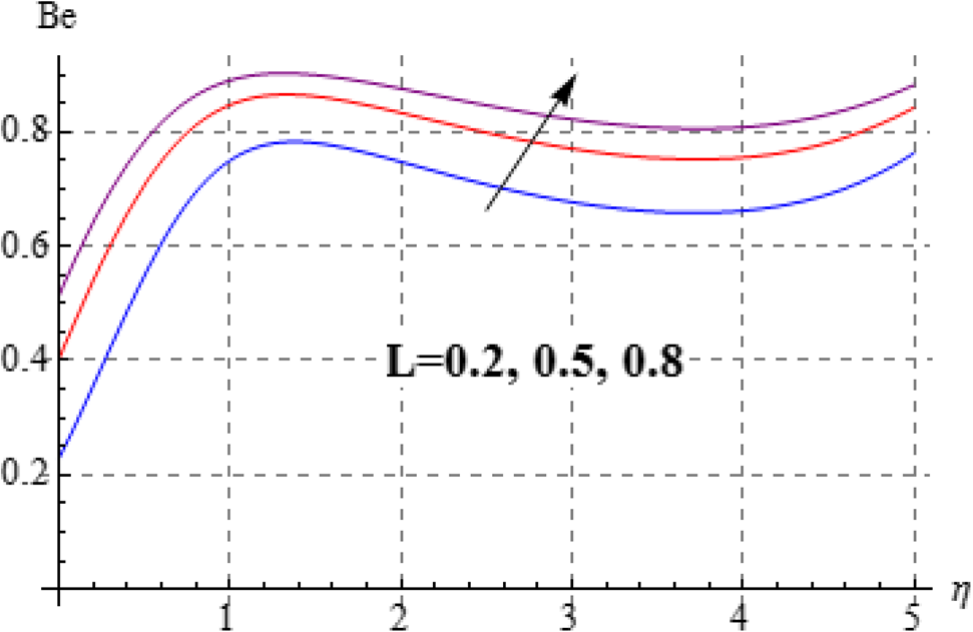
$$N_{G}$$ against L.

**Figure 21:**
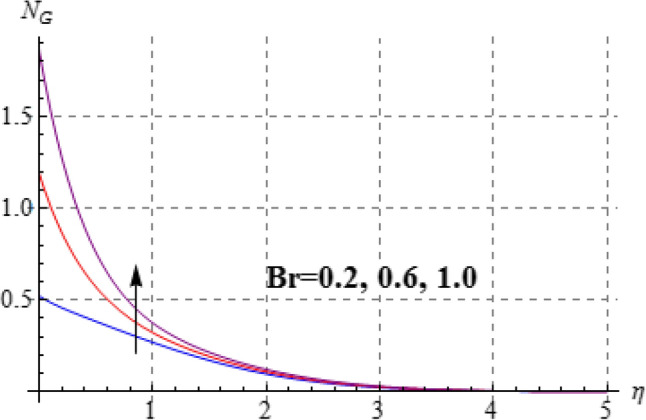
$$N_{G}$$ against Br.

**Figure 22:**
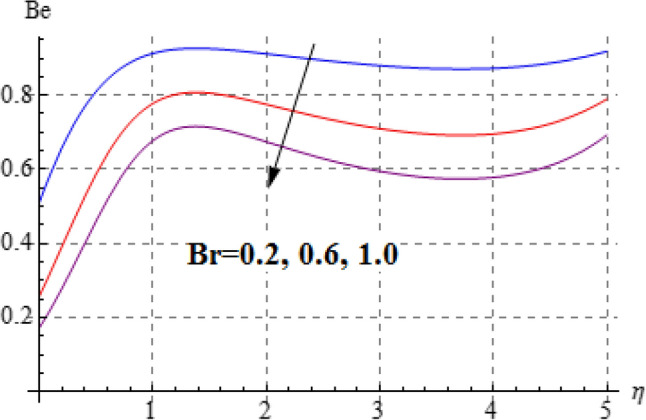
$$N_{G}$$ against Br.

Figures 15, 16, 17, 18, 19, 20, 21 and 22 are devoted to scrutinize the behaviors of various interesting parameter like viscosity parameter $\left( A \right)$, thermal conductivity parameter $\left( \delta \right)$, diffusion parameter $\left( L \right)$ and Brinkman number $\left( {Br} \right)$ on $Be$ and $N_{G}$. Figures 15 and 16 are depicted to explore the effect of $\left( A \right)$ on $Be$ and $N_{G}$. Here $N_{G}$ and $Be$ have opposite impact for increasing values of $\left( A \right)$. Variation of $\left( \delta \right)$ on $N_{G}$ and $Be$ is shown in Figs. 17 and 18. Clearly increasing values of $\left( \delta \right)$ give rise to both the $\left( {N_{G} } \right)$ and $\left( {Be} \right)$. Figures 19 and 20 are devoted to see the behavior of $\left( L \right)$ on $Be$ and $N_{G}$. Clearly for larger $\left( L \right)$ both $Be$ and $N_{G}$ have increasing behaviors. Figures 21 and 22 display impact of $\left( {N_{G} } \right)$ and $\left( {Be} \right)$ for Brinkman number $\left( {Br} \right)$. Larger Brinkman number rises the entropy generation. Figure 22 shows that for rising values of $\left( {Br} \right)$ the $\left( {Be} \right)$ decays.

### Analysis for engineering quantities

Here impacts of various influencing variables on gradient of velocity ($C_{fy}$ and $C_{fx}$) along azimuthal and tangential direction respectively, mass transfer rate $\left( {Sh_{x} } \right)$ and gradient of temperature $\left( {Nu_{x} } \right)$ are discussed in Tables 3, 4, and 5.

**Table 3:** Computational outcomes of $\left( {C_{fx} {\text{ and }}C_{fy} } \right)$.

$\lambda$	$A$	Surface drag force	
		$C_{fx}$	$C_{fy}$
1	0.2	1.1345	0.46536
3		2.02356	0.76543
5		3.0145	1.45362
2	0.2	0.89654	0.80983
	0.4	0.75643	0.69954
	0.6	0.65874	0.56432

**Table 4:** Computational outcomes of $\left( {Nu_{x} } \right)$.

$Br$	$\Pr$	$A$	$\delta$	$Nu_{x}$
0.0	0.7	2.0	0.2	0.6126
0.5				0.5325
1.0				0.4765
1.0	1.0	2.0	0.2	1.6875
	3.0			1.7894
	5.0			1.9283
1.0	0.7	1.0	0.2	0.7865
		2.0		0.6923
		3.0		0.6198
1.0	0.7	2.0	0.2	0.7967
			0.4	0.6987
			0.6	0.6089

**Table 5:** Numerical value of $\left( {Sh_{x} } \right)$.

$N$	$\gamma$	$Sc$	$Sh_{x}$
0.0	0.4	0.1	0.56796
0.5			0.60289
1.0			0.64156
0.5	0.1	0.1	0.45342
	0.3		0.49786
	0.5		0.53675
0.5	0.4	0.2	0.5745
		0.5	0.6745
		0.8	0.7981

#### Velocity gradient

The numerical results of $\left( {C_{fx} {\text{ and }}C_{fy} } \right)$ via various interesting parameters like viscosity parameter $\left( A \right)$ and mixed convection parameter $\left( \lambda \right)$ are analyzed in Table 3. Clearly one can find that an increment occurs in $\left( {C_{fx} {\text{ and }}C_{fy} } \right)$ via increasing values of $\left( \lambda \right)$. From this table it is noted that for larger estimation of viscosity variable the $\left( {C_{fx} {\text{ and }}C_{fy} } \right)$ are decreased.

#### Temperature gradient

Influences of different sundry variables like $\left( {Br} \right)$, $\left( {\Pr } \right)$, $\left( \delta \right)$ and $\left( A \right)$ on $\left( {Nu_{x} } \right)$ is scrutinized in Table 4. Nusselt number in enhanced for larger $\left( {Br} \right)$ and $\left( {\Pr } \right)$. Further $\left( {Nu_{x} } \right)$ is decreased for higher viscosity parameter $\left( A \right)$ and thermal conductivity parameter $\left( \delta \right)$.

#### Sherwood number

The computational outcomes of $\left( {Sh_{x} } \right)$ via various flow variables are studied in Table 5. Here $\left( {Sh_{x} } \right)$ has similar characteristics for larger $\left( N \right)$ and $\left( \gamma \right)$. We noticed that $Sh_{x}$ rises via $\left( {Sc} \right)$.

## Conclusions

The applications of entropy generation phenomenon in the convective transport of viscous nanofluid due to rotating cone have been addressed in presence of viscous dissipation and heat generation. The analysis is performed in presence of variable thermal conductivity and fluid viscosity. The key observations are given below.

- The tangential velocity and azimuthal velocity have contradictory behavior for mixed convection parameters.
- The applications of viscosity parameter show increasing effects on tangential velocity.
- The tangential velocity boosts up via buoyancy ratio variable.
- The nanofluid temperature is enhanced for larger heat generation variable it decreased for viscosity parameter.
- The nanofluid concentration is decreased for higher values of chemical reaction variable and Schmidt number.
- The entropy rate and Bejan number are enhanced for diffusion variable.
- The entropy rate upsurges versus Brinkman number.
- The entropy rate and Bejan number have reverse effects for viscosity parameter.
- The wall shear force increase via higher mixed convection parameter.
- The surface drag force is diminished against viscosity parameter as it is reversely related to the magnitude of drag force per unit area.
- The Nusselt number is increased for larger Prandtl number.
- Gradient of temperature versus Brinkman number decreases.
